# Cerebro-Spinal Meningitis—Its Pathology and Treatment

**Published:** 1873-05

**Authors:** J. G. Westmoreland

**Affiliations:** Atlanta, Georgia, Professor of Materia Medica in the Atlanta Medical College


### CEREBRO-SPINAL MENINGITIS—ITS PATHOLOGY AND TREATMENT.

BY J. G. WESTMORELAND, M.D., ATLANTA, GEORGIA,

*Professor of Materia Medica in the Atlanta Medical College.*

On the pathology of no disease so long known to the profession, of so frequent occurrence, and so fatal in its termination, have medical men differed more widely. The modes of treatment instituted upon these opposite opinions of the radical lesion or disturbance are, of course, dissimilar in every essential particular. This is not for the want of uniformity in the main features of the disease, as seen at different times, localities, etc., for while individual cases may differ from each other in degree and location, leading to varied local and general manifestations, yet in every instance the same character of symptoms and post-mortem appearances prevail.

The most important questions that present themselves, in the investigation to determine whether or not cerebro-spinal meningitis be a misnomer for the disease in question, are: 1. Do autopsies of undoubted cases invariably present evidences of some stage of the inflammatory process in the meninges; and 2. Do the symptoms and result of treatment justify this opinion?

The doctrine of its non-inflammatory character seems to have originated in the fact that instances of fatal termination in a few hours sometimes occur, even before the imflammatory process is thoroughly established, or at any rate before sufficient time has elapsed for its usual destructive results to supervene. This has impressed certain pathologists with the idea of a subtle poison affecting the nervous centres, and that their functions are suspended as by the action of other narcotic poisons.

Now, we take the position that the disease has been properly named; that ordinary acute inflammation exists in parts indicated by the name, and that death, when it occurs, is the result of this process. While this opinion is entertained, we are not ignorant of the fact that the disease occurs under peculiar circumstances when it prevails extensively. Epidemic influence seems to favor its development. *Influenza*, signifying influence, is an unmeaning term, used as a kind of generic name for the various local inflammatory diseases that occur during its prevalence. This epidemic influence, or influence upon the people, as the term implies, differs from those epidemics which lead to the development of dysentery, typhoid fever, etc., in the character of the lesion and the parts attacked. Narritis, laryngitis, tonsilitis and bronchitis are the results of the former influence or influenza, and when slight, take the name of catarrh or bad cold; but when protracted, affecting the eustachian tube, auditory apparatus and antrums, it is called influenza. Under the same kind of influence at other times, and perhaps even the same season, the lungs, pleura and meninges of the brain and spinal cord are subject to inflammatory action, which takes the common names of pneumonia, pleurisy, and meningitis. And while local inflammation occurs in these parts under this epidemic, the alimentary canal suffers in epidemic bowel affections, and are named rectitis, colitis, colo-rectitis or enteritis, according to the part inflamed.

Sporadic cases of any of the above-named local inflammatory diseases may occur at any time without epidemic influence, from sudden lowering of the temperature when high, or other ordinary cause deranging the circulation and nervous system. These differ from epidemic in degree and time of duration, but not in character.

Now acute inflammation, whether occurring sporadically or from an influence upon the people generally, should be named and treated promptly as such. It must, of course, be understood that spanæmic or other depressing influences being exerted upon patients at the same time—such as malaria, zymotic tendencies, etc.—should modify the treatment decidedly.

To the argument that speedy termination of meningitis in death disproves the theory of its inflammatory character, we answer that the extensive and violent inflammation (not poison) shocks the nervous centres, so as to depress the vital energies, as from violent traumatic injury; and also that engorgement—the first stage of inflammation—is sufficient alone, when suddenly produced, as in congestive apoplexy or first stage of meningitis, to arrest the functions of nervous centres, by mechanical pressure, without any such shock.

By the following statistics of treatment and post-mortem examinations, we propose to show that meningitis is a highly inflammatory disease connected with vital parts, and should be treated promptly and actively. The cases are not selected, but all reported were treated from date of the first to that of the last:

Case I.—A girl aged six years was attacked in the afternoon of February 10th, with the ordinary symptoms attendant upon the rise of fever from any cause, the pain in the head and back of the neck being excessive. During the night, vomiting with rigidity of the neck; slight delirum and muscular agitation approaching spasm occurred.

Saw her first at 10 o'clock on the 11th—eighteen hours after attack—and found her in very much the condition above described. No decided opisthotonos, but stiffness with tenderness of the neck was present; vomited occasionally; pulse one hundred and twenty or thirty, and of good force and volume.

The treatment was commenced by the administration of one-twelfth grain of morphine and the abstraction of six or eight ounces of blood from the arm. Ffteen grains of calomel were given, and a blister applied to back of the head and neck. No more vomiting occurred, and although febrile excitement continued for several days, the nervous agitation, pain and stiffness gradually subsided. A week from the attack all the unpleasant symptoms had disappeared, except periodical rise of fever, recurring daily, for which quinine was given successfully.

Case II.—A young man, aged twenty-three, came to town from the country, (Gwinnett county,) March 18th, 1872, and in the afternoon of that day complained of general indisposition, with chilly sensation.

The 19th, in the morning, he was seen walking about the hotel, but evidently with the mental and physical energies obtunded. He went to his room, and was found at 10 o'clock in a stupid and almost pulseless condition. I saw him about 10½ o'clock, and found the skin cool; pulse about ninety, small and very feeble; neck stiff and tender to the touch, and head drawn back; was aroused with difficulty, and had been vomiting considerably. One-fourth grain acetate morphia was given, and blood abstracted from temples, back of head and neck, to the amount of two or three ounces, and from a vein in the arm about four ounces, requiring pressure or stripping of the hand and arm to obtain this amount. Blister applied to occiput and neck. Forty grains of calomel were given, and I left the patient at 12½ o'clock, with increased temperature of the skin, more vigorous circulation and less stupor.

Returned at 2 P.M., and found reaction perfect; pulse a little more frequent, sufficiently full and strong; no more vomiting; excessively restless, talking incoherently, tossing from side to side, and not remaining in the same position more than two minutes at a time. A vein in the arm was again opened, and sixteen ounces taken, greatly to the relief of restlessness and delirium.

No other special treatment was used, except the occasional use of opium and reapplication of the blister. The fever, and more or less delirium, with rigid muscles of the neck, continued for about ten days, when he commenced slowly to convalesce, was carried home and recovered perfectly.

CASE III.—A girl, aged five years, was attacked with chill and pain in the head, at 10 A.M. December 1, 1872. I saw her at 2 P.M., and found her inclined to sleep, but countenance anxious when awake, and complaining of pain in back of head and neck, with general headache and fever. No decided stiffness of the neck nor delirium were observable, and while fears of meningeal trouble were entertained, I left her with directions to give ten grains of calomel, and apprise me immediately on appearance of any more unpleasant symptom. I was again called in haste at 6 P.M., and learned that she had a convulsion half an hour previously. Found her still inclined to sleep, but less at ease when awake. No opisthotonos, but vomiting and muscular agitation with slight delirium when first aroused. Not being entirely satisfied as to her true condition, and thinking it better to err on the safe side, blood was abstracted from the arm to the amount of three or four ounces, five grains more of calomel given, and a blister applied to back of head and neck. Another convulsion came on about 8 P.M., but no further serious trouble during the night. The blister drew well, and no further treatment became necessary, nor was anything given, except some paregoric, after the means already mentioned. The patient was well in a few days.

CASE IV.—A boy, aged sixteen years, was seen first on March 26, 1873, at 8 A.M. The history was that during the afternoon of the 24th, he had complained of pain and stiffness of the neck, a general aching of the limbs with chilliness. On the 25th, in the morning, the symptoms were aggravated, but he had walked out in town to consult a physician. He was delirious and sleepless.

Found him delirious, excessively restless, with complete opisthotonos, pupils dilated, complaining of pain in the head and back of the neck, when a rational answer could be had. The head was so drawn back that, in whirling over from side to side, which was constant, he was compelled to rotate upon his face. Pulse about eighty, and nearly normal in force of volume. The treatment consisted of blood-letting from the arm to the amount of fifteen ounces, one-quarter grain morphine, the abstraction of about five ounces of blood by cups from the head and neck, the administration of forty grains calomel, and the application of a blister to back of head and neck. During the flow of blood from the arm he fell asleep, which he had not done for twenty-four hours. The neck, though still firmly fixed, was not so far drawn back after a few hours. The delirium, with all other unpleasant symptoms, gradually subsided, and in five or six days was convalescent, without further treatment other than an opiate once or twice a day, and one saline cathartic about the fourth day. His restoration to health was perfect in every respect.

Case V.—A girl, aged ten years, visited at 4 p.m. on March 25, 1873, was complaining of lassitude, chilliness and aching of the limbs during the morning, until 10 o'clock, when high febrile excitement was established, with severe pain in the head and back of the neck, pupils dilated. Total blindness of the left eye came on, which lasted for an hour or two, when every object seen with that eye appeared quadruple, and sometimes five objects were seen where only one existed. This peculiar perturbation of the function of the left eye existed when seen on the first visit, at 4 o'clock, with nausea, pain and soreness of the neck and some stiffness, but no very distinct opisthotonos. The mind clear, skin warm, and pulse one hundred and twenty, firm and full. About one-tenth grain of acetate morphia was given, and six ounces of blood taken from the arm, when the vision became perfect and the pain less. Three hours afterward one or two ounces of blood were obtained by cups from back of the neck, eighteen grains of calomel administered, and a blister applied to back of head and neck. Without other treatment the fever and pain gradually subsided, and the patient was convalescent in two days.

The blood in this case attracted my attention, especially. The coagulation was speedy, and so perfectly tough and tenacious, without the appearance of serum, that it was with difficulty washed from the vessel.

The following cases were furnished by a professional brother, of this city, and are not selected, but include all treated from date of the first case, and are perfectly reliable:

Case I.—Mulatto female, aged twenty-six years. Called at

2 A.M. in November, 1872, and found her moribund. History: Was attacked three days before, after exposure, with violent pain in head; intolerance of light and sound; constipation, vomiting; hyperæthesia of the skin; stiffness of the neck, and convulsions; unconscious; one pupil dilated, the other contracted; jaws firmly closed. Had received no treatment, and died at 4 A.M., two hours after first seen.

CASE II.—White man, aged twenty years. First seen November 28th. Presented symptoms of acute articular rheumatism. Had for two days previously suffered with intense pain in head and neck, which had subsided. Two days later, pain in head returned; pulse irregular; constipation; slight nausea and hyperæsthesia of the skin and throat; gradually the head was drawn back; intolerance of light and noise; temperature one hundred.

Treatment: Quinine and fluid extract ergot, three times a day; opium and occasional laxative. Occasional slight delirium attended the case till he died, December 24th.

Autopsy, eighteen hours after death: Meninges of brain intensely congested, and considerable effusion. A thick exudation covered left and part of right lobe of cerebellum, also enveloping medulla and spinal cord. Substance of brain seemed healthy, but pia mater injected, and at points broken down in texture.

CASE III.—Boy, aged fourteen years. Attacked early Friday morning, March —, with violent pain in head and neck and vomiting; intolerance of light and noise; neck stiff and slightly drawn back; constipated; pulse one hundred; temperature one hundred.

Treatment: Fifteen grains calomel; blister to spine; four grains quinine and one-quarter grain opium, every four hours. Sunday, one grain ergotine, hypodermically four times. Monday, better. Dismissed well in a week from attack.

CASE IV.—Boy, aged six years. Attacked three days before first visit, April 5th; had received no treatment. Symptoms as in case three, but more violent; pulse, one hundred and forty; respiration, thirty-six; head drawn violently back.

Treatment: Ten grains calomel, four drops laudanum and two grains quinine, every four hours; one-half grain ergotine three times a day. Continues to sink, and will probably die soon.

